# The impact of increasing income inequalities on educational inequalities in mortality - An analysis of six European countries

**DOI:** 10.1186/s12939-016-0390-0

**Published:** 2016-07-08

**Authors:** Rasmus Hoffmann, Yannan Hu, Rianne de Gelder, Gwenn Menvielle, Matthias Bopp, Johan P. Mackenbach

**Affiliations:** Department of Public Health, Erasmus Medical Center, P.O. Box 2040, 3000 CA Rotterdam, Netherlands; Sorbonne Universités, UPMC Univ Paris 06, INSERM, Institut Pierre Louis d’épidémiologie et de Santé Publique (IPLESP UMRS 1136), F75012 Paris, France; Epidemiology, Biostatistics and Prevention Institute, University of Zurich, Zurich, Switzerland

**Keywords:** Income inequality, Health inequality, Mortality, International comparison, Fixed-effects, Longitudinal analysis, Europe

## Abstract

**Background:**

Over the past decades, both health inequalities and income inequalities have been increasing in many European countries, but it is unknown whether and how these trends are related. We test the hypothesis that trends in health inequalities and trends in income inequalities are related, i.e. that countries with a stronger increase in income inequalities have also experienced a stronger increase in health inequalities.

**Methods:**

We collected trend data on all-cause and cause-specific mortality, as well as on the household income of people aged 35–79, for Belgium, Denmark, England & Wales, France, Slovenia, and Switzerland. We calculated absolute and relative differences in mortality and income between low- and high-educated people for several time points in the 1990s and 2000s. We used fixed-effects panel regression models to see if changes in income inequality predicted changes in mortality inequality.

**Results:**

The general trend in income inequality between high- and low-educated people in the six countries is increasing, while the mortality differences between educational groups show diverse trends, with absolute differences mostly decreasing and relative differences increasing in some countries but not in others. We found no association between trends in income inequalities and trends in inequalities in all-cause mortality, and trends in mortality inequalities did not improve when adjusted for rising income inequalities. This result held for absolute as well as for relative inequalities. A cause-specific analysis revealed some association between income inequality and mortality inequality for deaths from external causes, and to some extent also from cardiovascular diseases, but without statistical significance.

**Conclusions:**

We find no support for the hypothesis that increasing income inequality explains increasing health inequalities. Possible explanations are that other factors are more important mediators of the effect of education on health, or more simply that income is not an important determinant of mortality in this European context of high-income countries. This study contributes to the discussion on income inequality as entry point to tackle health inequalities. More research is needed to test the common and plausible assumption that increasing income inequality leads to more health inequality, and that one needs to act against the former to avoid the latter.

**Electronic supplementary material:**

The online version of this article (doi:10.1186/s12939-016-0390-0) contains supplementary material, which is available to authorized users.

## Background

In many European countries health inequalities are increasing [1–5]. Likewise income inequality is increasing in many but not all European countries [6, 7]. In this paper, we test the hypothesis that these two trends are related, i.e. that countries with a greater increase in income inequalities have also experienced a greater increase in health inequalities, and that increasing income inequalities can explain some of the widening of health inequalities over time.

The plausibility of this hypothesis can be derived in two different ways. First, higher income inequality has been associated with lower life expectancy and other health measures [8]. This finding has been discussed and researched intensively in the past 20 years [9–14], and one of the possible explanations for such an association is that higher income inequality leads to larger health inequalities [12]. While many different mechanisms may be involved in the hypothesized effect of income inequality on health on the social level, our empirical study focuses on a second, more straightforward explanation: if income partly mediates the effect of education on mortality, one would expect countries with larger income inequalities between educational groups also to have larger mortality inequalities between educational groups. Income is associated with mortality [15, 16], as well as with other health outcomes [17–19], probably because it is needed to buy healthy food, good housing in a safe environment, quality health care, etc. [20–23]. Also, there is a strong association between education and income, partly because higher education provides better opportunities on the labour market [24]. As a result, income (or material conditions more generally) has indeed been found to partly mediate the effect of education on mortality [25–28].

Our hypothesis that income differences between educational groups predict mortality differences between educational groups has not been directly tested as yet, but previous studies have found results inconsistent with our hypothesis. Studies comparing the degree of health inequality between countries that have different degrees of income inequality have surprisingly revealed that countries with smaller income inequalities, such as the Scandinavian countries, often have larger mortality inequalities (measured by education or income) than countries with larger income inequalities, such as the USA or Mediterranean countries [25, 29–33]. On the other hand, countries with larger income inequalities usually have larger inequalities in self-assessed health between income groups [17].

However, all these studies were based on cross-sectional international correlations, and are therefore inconclusive with regard to causality. To address this problem, our study looks at changes over time in income inequality within countries, and assesses whether these are associated with changes in inequality in mortality. To the best of our knowledge, only two studies exploit changes over time. One is a study comparing trends in occupational class differences in material living standards to trends in occupational class differences in mortality in England & Wales, which found that trends in relative poverty did explain some of the changes in inequality in mortality [34]. However, as this was a single-country study, the scope for causal inference was limited. The other is an international time-series analysis, in 34 North American and European countries, of socioeconomic inequalities in adolescent health as the outcome of, among others, changes in income per person and income inequality (GINI). The authors found the expected association between social inequality (measured by material indicators) and health inequality [35]. Data from Scandinavian countries suggests a lack of association between trends of income inequality and health inequality measured by several indicators for social stratification [36]. In the period from the 1970s to the mid-1990s, inequality in mortality increased in Denmark, Finland, Norway and Sweden [37] while income inequality was stable, at least until the early 1990s in Denmark and Finland. In Norway and Sweden, income inequality increased only after the mid-1980s, after the most rapid increases in health inequality [38].

Our study tests the common and plausible claim that increasing income inequality will lead to more health inequality, and that one needs to act against the former to avoid the latter [39–43] – a claim which has not been universally accepted [36, 44].

## Methods

We selected countries for which comparable data on mortality, as well as income by education, were available for the same time periods (or slightly later for mortality than for income) and for at least two points in time. Our study covers six countries, representing different European regions: Belgium, Denmark, England & Wales, France, Slovenia, and Switzerland. Years of income measurement are shown in Table 1 and periods of mortality data are shown in Table 2.

**Table 1:** Description of the survey data

Country	Survey name	Survey years	Sample size	Low educated (ISCED 0–2, %)	Middle educated (ISCED 3–4, %)	High educated (ISCED 5–6, %)	Income measure	Income item non-response (%)
Belgium	Health Interview Survey	1997	6288	40.5	30.3	29.3	net	4.9
		2001	7640	40.0	29.4	30.6		13.1
		2004	7811	40.0	29.1	30.9		14.5
Denmark	Danish Health and Morbidity Survey	1994	3322	30.8	54.2	14.9	gross	9.8
		2000	12373	26.3	54.8	18.8		9.3
		2005	11469	21.9	55.5	22.6		7.9
England&Wales	General Household Survey	1990	10369	58.0	25.4	16.5	gross	16.2
		1996	9961	49.5	29.1	21.4		17.3
		2000	9121	33.2	39.2	27.6		16.4
		2005	14323	34.0	37.5	28.6		15.5
France	Health Barometer	2000	9641	33.3	41.1	25.7	net	5.4
		2005	20105	27.6	41.6	30.7		12.5
Slovenia	Slovenian Public Opinion Survey	1994 + 1996	1012	37.4	52.3	10.3	net	28.4
		1999 + 2001	1035	28.9	57.0	14.1		33.0
Switzerland	Swiss Health survey	1997	8267	19.8	63.1	17.0	net	5.8
		2002	14075	16.8	66.2	16.9		3.9
		2007	12878	13.2	61.6	25.2		4.8

**Table 2:** Number of deaths and person-years for all-cause and cause-specific mortality, by country, gender, educational group and period

	All deaths	CVD	Cancer	External	Other	Person-years	All deaths	CVD	Cancer	External	Other	Person-years	All deaths	CVD	Cancer	External	Other	Person-years
Belgium	1996–2000						2001–2006						2007–2009					
low educated (M)	118421	NA	NA	NA	NA	6407982	105757	NA	NA	NA	NA	5970453	60504	NA	NA	NA	NA	3554647
high educated (M)	13469	NA	NA	NA	NA	1964531	18925	NA	NA	NA	NA	3200120	12922	NA	NA	NA	NA	2143056
low educated (F)	79799	NA	NA	NA	NA	7605202	79353	NA	NA	NA	NA	6769775	44294	NA	NA	NA	NA	3995676
high educated (F)	6484	NA	NA	NA	NA	1769456	8854	NA	NA	NA	NA	3094898	6632	NA	NA	NA	NA	2151643
Denmark	1991–1995						1996–2000						2001–2005					
low educated (M)	82260	34043	23803	3888	20526	2879895	65457	23445	19275	3236	19501	2619922	48692	15620	15282	2269	15521	2371357
high educated (M)	6230	1963	2187	611	1469	970282	8514	2519	3234	555	2206	1168456	11211	3322	4287	604	2998	1406524
low educated (F)	72760	27067	23675	3067	18951	3930208	62915	19759	21263	2405	19488	3535190	49941	13801	17779	1372	16989	3062525
high educated (F)	3417	540	1837	330	710	893934	4937	794	2584	359	1200	1155526	6887	1371	3308	356	1852	1527771
England&Wales	1991–1995						1996–2000						2001–2005					
low educated (M)	7823	3581	2565	177	1500	470395	7072	3006	2303	191	1572	453846	5169	1865	1857	162	1285	338033
high educated (M)	789	355	274	29	131	111191	818	325	314	32	147	115782	769	263	274	47	185	131316
low educated (F)	6175	2468	2222	125	1360	547626	5694	2077	2045	107	1465	523884	4307	1410	1600	98	1199	401301
high educated (F)	366	113	167	23	63	82117	364	113	163	11	77	87852	490	117	230	19	124	129356
England&Wales	(continued) 2006–2009																	
low educated (M)	3050	1026	1084	82	858	235349												
high educated (M)	551	177	239	22	113	101360												
low educated (F)	2693	727	1086	53	827	276224												
high educated (F)	405	87	191	12	115	100753												
France	1999–2003						2004–2007											
low educated (M)	4066	1012	1670	286	1098	232761	2766	629	1159	214	764	161535						
high educated (M)	408	81	193	33	101	99336	406	87	179	41	99	84549						
low educated (F)	2600	677	978	160	785	333404	1925	453	807	102	563	231945						
high educated (F)	175	21	100	19	35	101300	175	20	105	16	34	89985						
Slovenia	1996–2001						2002–2006						2007–2011					
low educated (M)	38151	13924	11538	N/A	12689	1470820	16598	5506	5186	N/A	5906	724842	17639	6044	5922	N/A	5673	637051
high educated (M)	3501	1344	1264	N/A	893	275375	3216	1077	1218	N/A	921	371373	4034	1304	1714	N/A	1016	352609
low educated (F)	36149	16727	9188	N/A	10234	1896579	17228	7045	5181	N/A	5002	1205125	23038	10602	6474	N/A	5962	1101551
high educated (F)	1157	319	559	N/A	279	209281	1220	280	659	N/A	281	366379	1707	453	866	N/A	388	358818
Switzerland	1996–2000						2001–2005						2006–2008					
low educated (M)	24851	8622	7149	1359	7721	1078295	18448	5805	5614	1118	5911	1038023	9248	2818	3012	596	2822	544751
high educated (M)	11185	3642	3551	970	3022	1553571	10897	3120	3751	1031	2995	2138595	6610	1783	2458	596	1773	1297871
low educated (F)	28105	9602	8647	1035	8821	2718754	20986	5940	7241	960	6845	2419220	11091	2746	4214	454	3677	1276367
high educated (F)	1920	379	829	135	577	539677	2357	374	1084	228	671	889636	1485	225	727	132	401	559904

We calculated rates for all-cause and cause-specific mortality for four causes of death: cardiovascular diseases (CVD) (code of the 10th Revision of the International Classification of Diseases I00-I99), cancer (C00-D48), external causes (V01-Y98) which are mainly composed of deaths from accidents, violence, and suicide. Our mortality data covered the whole population, except for England & Wales and France, where a 1 % representative sample was used. Mortality data stemmed from longitudinal mortality follow-up after a census and socioeconomic information of the population-at-risk came from the census. The data was provided by national statistical offices. As indicator of socioeconomic position, we used self-reported level of education, using the ‘low’ and ‘high’ categories corresponding to the International Standard Classification of Education (ISCED 1997) categories 0–2 and 5–6 [45], leaving out the mid-educated. Mortality rates by educational level were age-standardised using the European Standard Population [46]. All analyses were restricted to the age-range 35–79 years, because below the age of 35 people may still be studying for a degree (and because most health problems are rare before that age), and because above the age of 80 the proportion of institutionalized people increases strongly, and institutionalized people are excluded from most surveys. Available mortality data for Belgium did not include cause-specific information, thus Belgium was left out of the cause-specific analyses. Slovenia could not provide mortality data for external causes of death. Numbers of deaths and person-years can be found in Table 2.

Social inequalities in mortality and income were measured by calculating absolute (rate differences, RD) and relative (rate ratios, RR) differences in these indicators between low and high educated persons, leaving out the middle educated. It is important to look at both relative and absolute inequalities, because these two perspectives can reveal quite different changes over time, depending on the underlying trend of income or mortality. Income is measured as the gross or net equivalent income, weighting the first person of the household by 1 and all other persons by 0.5. While for most countries and years income was measured as a continuous variable, Denmark used 12, 17 and 13 income categories (in 1994, 2000 and 2005 respectively), England & Wales used 21 categories in 1990 and then adopted a continuous measurement, and France used 12 categories. This categorical data was translated into continuous amounts by taking the midpoint of each income category. For the highest and open category an algorithm was applied that takes into account the overall income distribution. The trends of income inequalities for England & Wales showed no jumps between years in which categories were used (until 1990) and years with continuous measurement (1996 onwards), suggesting that a high number of income categories limits the bias due to the categorical measurement. In order to compare absolute income differences across periods and countries, the given amounts from the survey data have been adjusted by purchasing power parities (PPP) by the OECD [47] to be interpretable as comparable US dollars. This adjustment was not done for the analysis of relative income inequalities.

All analyses were done separately by gender. As a first preparatory step, we established the trend for all absolute and relative inequality measures (income, all-cause mortality, cause-specific mortality) for each country to see where we find statistically significant trends. The sample size of our unbalanced time series data is 18 data points for income and 17 data points for mortality. The main analysis used fixed-effects panel regression models including dummy variables for each country, first, to determine the average trend of income inequalities for all countries together (Model 0), second, to determine the average trend of mortality inequalities for all countries together (Model 1) and, finally, to see if the average trend in income inequality predicted the overall change in mortality inequality (Model 2). Fixed-effects models allow taking time-invariant unknown country characteristics into account that would otherwise bias the results. Clustered sandwich estimators were used to allow for within-country correlation between error terms. Our models are represented by the following equations, where *incomeinequality*_*it*_ is the income inequality of country *i* in year *t*, *mortalityinequality*_*it*_ represents the mortality inequality of country *i* in year *t*, *α* is a constant, *year* denotes the continuous time trend, *country*_*i*_ is a vector of country dummies, and *e* is the error term.

$$ \left(\mathrm{Model}\ 0\right)\kern1.1em  incomeinequalit{y}_{it}=\alpha +\beta yea{r}_t+ countr{y}_i+{e}_{it} $$

$$ \left(\mathrm{Model}\ 1\right)\kern1em  mortalityinequalit{y}_{it}=\alpha +\beta yea{r}_t+ countr{y}_i+{e}_{it} $$

$$ \left(\mathrm{Model}\ 2\right)\kern1em  mortalityinequalit{y}_{it}=\alpha +\beta yea{r}_t+ Yincomeinequalit{y}_{it}+ countr{y}_i+{e}_{it} $$

## Results

**Fig. 1:**
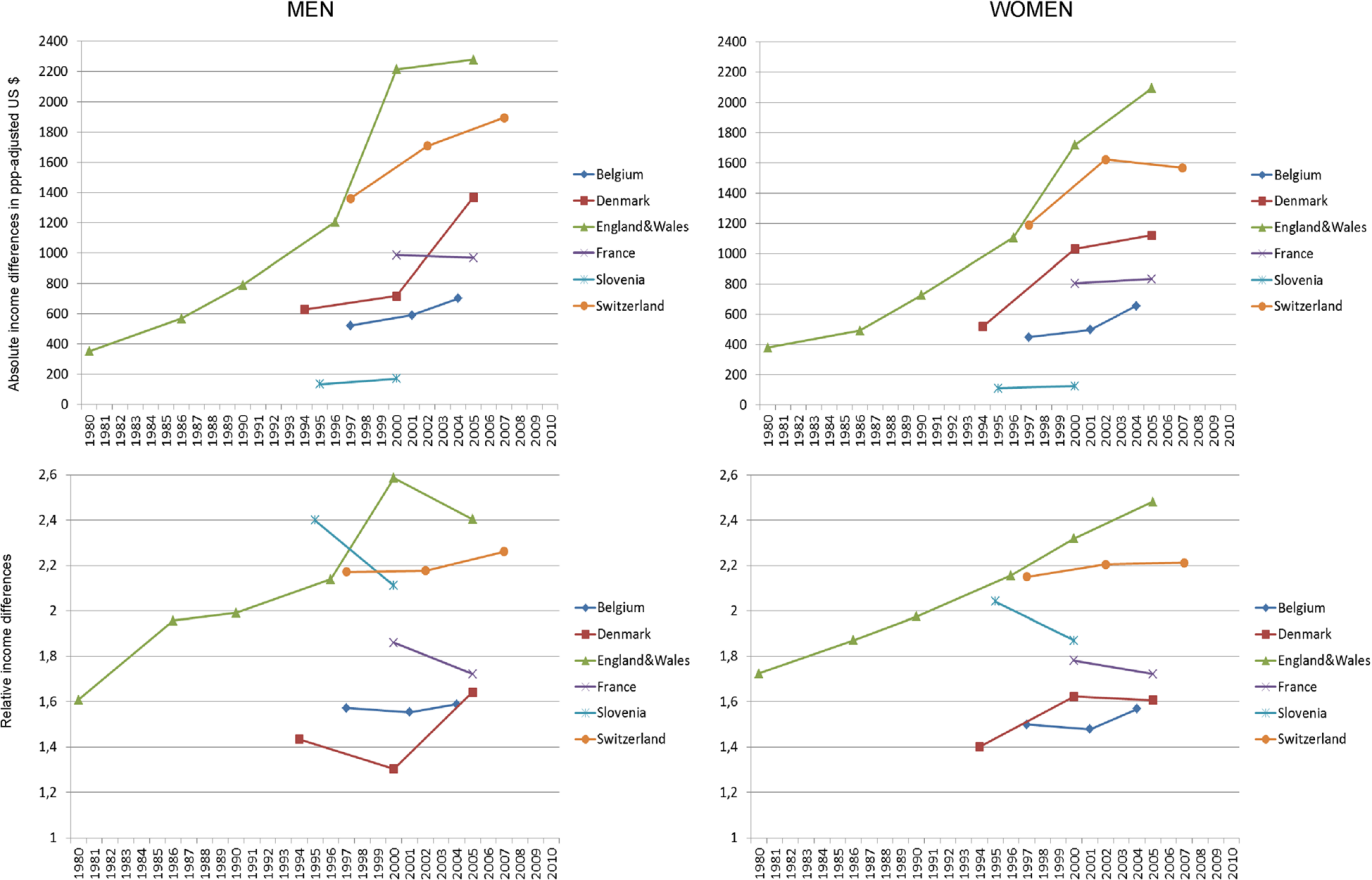
Trends in income inequality between low and high educated people in six European countries. Note: The first two data points for England & Wales are shown in Fig. 1 but not used in the regression analyses in order to keep the period of analysis similar across countries

**Fig. 2:**
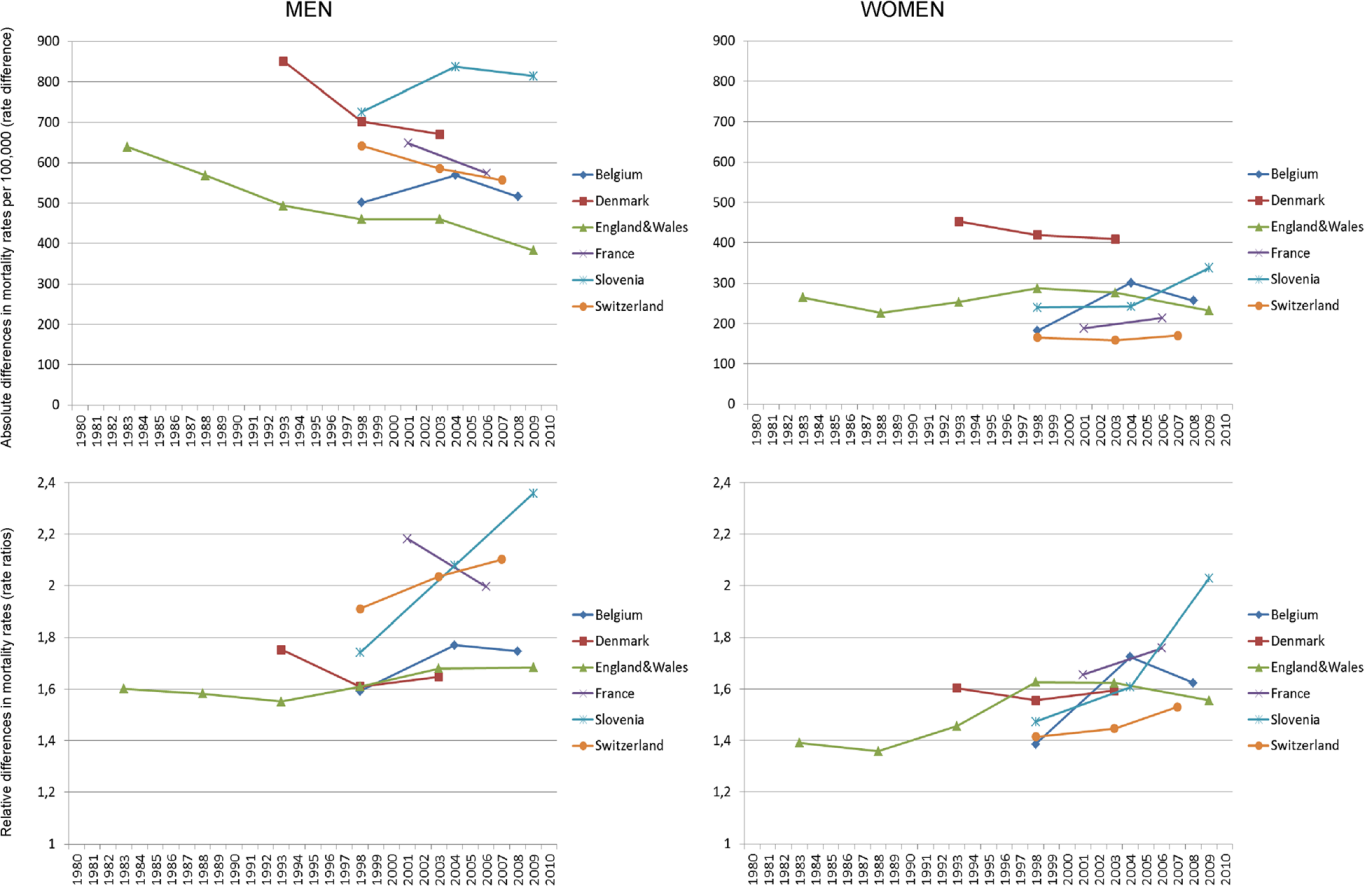
Trends in mortality inequality between low and high educated people in six European countries. Note: The first two data points for England & Wales are shown in Fig. 2 but not used in the empirical analysis in order to keep the period of analyses similar across countries. Rates are per 100,000

Figure 1 shows that absolute income inequality between educational groups generally increased over the study period. However, this is not a universal phenomenon, as France and Slovenia showed almost no increase. Trends in relative income inequality revealed a more diverse international pattern, with England & Wales clearly increasing, France and Slovenia clearly decreasing, and other countries showing variable trends. This difference between absolute and relative results can be explained by the fact that as income increases in many countries, absolute inequalities are more likely to increase than relative inequalities. Figure 2 shows that absolute inequality in mortality has decreased for men and has been stable for women. Relative inequality in mortality has increased for both men and women (with international differences), the only clear exception being men in France, among whom inequality decreased. Analogously to income inequality, the differences between absolute and relative trends can be explained by the fact that as mortality decreases in all countries, absolute inequalities are more likely to decrease even when relative inequalities increase. An important gender difference can also be seen in our mortality trends: mortality decline is stronger for men than for women.

Table 3 shows the country-specific annual changes in inequality in income and all-cause mortality as produced by regression models. This way of describing the trends goes beyond Fig. 1 by showing the level of statistical significance of the change. As in Figs. 1 and 2, income inequality mostly increased and absolute mortality differences decreased while relative mortality differences increased. The cause-specific results confirm this overall pattern, with the exception that absolute differences in mortality from cancer for women increased (see Additional file 1: Table S1).

**Table 3:** Annual changes of absolute and relative inequalities in income and all-cause mortality

	Income				All-cause mortality			
	Absolute inequality		Relative inequality		Absolute inequality		Relative inequality	
	Men	Women	Men	Women	Men	Women	Men	Women
Belgium	30.00*	34.61	0.29	1.15	2.27	8.45	1.67	2.63
Denmark	74.04	60.16	2.08	2.06	-18.06	-4.35	-1.05	-0.09
England&Wales	101.91*	88.60***	3.08	3.16***	-6.40*	-1.58	0.87*	0.52
France	-3.33	5.67	-2.75	-1.18	-14.86	5.18	-3.73	2.05
Slovenia	6.17	2.44	-4.81	-2.85	8.37	8.62	5.64***	5.00
Switzerland	59.77*	43.86	0.96	0.71	-9.59*	0.43	2.14*	1.26

The annual changes are the slope coefficients from linear regression models of the particular inequality measure on the variable “year”. Statistically significant results are printed in bold, significance levels are *:*p* < 0.1; **:*p* < 0.05; ***:*p* < 0.01. Slopes for relative differences have been multiplied by 100 to make them more legible: 1.0 means that relative inequality changes e.g. from 1.55 to 1.56 in one year. For absolute differences, e.g. a slope of 30.00 means that absolute income inequality increases by US$ 30 per year

We also used the data in Table 3 to see whether country-specific trends in income inequality were correlated with trends in mortality inequality. About half of all correlation coefficients were negative (including about half of those that were statistically significant). Negative correlations mean that increasing income inequality is associated with decreasing mortality inequality, or vice versa. All correlations between the trend in income inequality and the trend in mortality inequality were negative for all-cause mortality. Cause-specific correlation coefficients suggested that inequality in cancer mortality is negatively correlated and inequality in external causes of death is positively correlated with the trend in income inequality (see Additional file 2: Table S2).

In the next step, we combined the trends of inequalities for all countries by estimating regression models that identified overall trends using dummy variables for the country. This produced overall trends for income inequality (Model 0 in Table 4). Increases in income inequality were mostly statistically significant and similar for men and women, i.e. gender differences were not statistically significant. The results indicate that across all countries, absolute income differences between low- and high-educated people increased by $ 75.3 (men) and $ 64.3 (women) per year, respectively. Relative inequalities increased by almost two percentage points per year, which means, for example, an increase of income advantage of the high educated from 1.50 to 1.52.

**Table 4:** Regression models for annual trends in inequalities in all-cause and cause-specific mortality (Belgium, Denmark, England & Wales, France, Slovenia, Switzerland)

			Income	Total mortality		CVD		Cancer		External		Other	
		Coefficient for	Model 0	Model 1	Model 2	Model 1	Model 2	Model 1	Model 2	Model 1	Model 2	Model 1	Model 2
Absolute inequalities	men	year (annual trend)	75.3***	-4.95	-5.08	-0.01	8.30	2.19	2.02	-0.12	-0.52	1.60	3.25
		income inequality			-0.02		-0.13		-0.01		0.01		-0.01
	women	year (annual trend)	64.3***	1.67	4.48	-9.48	-26.05	2.96	5.03*	0.52	-0.11	1.84	3.27**
		income inequality			-0.07		0.27		-0.02		0.01**		-0.02
Relative inequalities	men	year (annual trend)	1.65	1.51	1.08	3.40	3.23	1.56	1.10	0.71	0.01	4.02**	3.87*
		income inequality			-0.08		-0.60		0.01		0.41		-0.39
	women	year (annual trend)	1.90*	1.64	1.72**	-3.86	-13.00*	1.70*	1.65**	2.30	1.05	2.76	4.22***
		income inequality			-0.36**		5.00*		-0.12		0.83*		-1.19*

Model 0 estimates a general trend of income inequality between high- and low-educated: *incomeinequality*_*it*_ = *α* + *βyear* + *country*_*i*_

Model 1 estimates a general trend of mortality inequality between high- and low-educated: *mortalityinequality*_*it*_ = *α* + *βyear* + *country*_*i*_

Model 2 estimates a general trend of mortality inequality, taking the trend of income inequality into account: *mortalityinequality*_*it*_ = *α* + *βyear* + *Υincomeinequality*_*it*_ + *country*_*i*_

Coefficients for income inequality in Model 0 mean that e.g. absolute income inequality increased by 75.3 US$ per year

Coefficients for the annual trend in Model 1 mean that e.g. absolute differences in total mortality decreased by 4.95 deaths per 100.000 per year

Coefficients for the annual trend in Model 2 mean that e.g. absolute differences in total mortality decreased by 5.08 deaths per 100.000 per year if the trend in income inequality is added to the model

Coefficients for income inequality in Model 2 mean that one-unit increase in income inequality leads to, e.g., a 0.02 unit decrease in inequality in total mortality. Statistically significant results are printed in bold, significance levels are *:p < 0.1; **:p < 0.05; ***:p < 0.01

Belgium was excluded from the cause-specific analysis because data was not available. Slovenia was excluded from the analysis of external causes because data was not available

Likewise, we ran such models for the overall trend in mortality inequality for all countries together (Model 1 in Table 4). In general, mortality trends were less clear than for income but they confirmed some findings in Fig. 2, although without statistical significance: absolute inequalities in all-cause mortality decreased among men, while they increased among women. Relative inequalities increased for both men and women. The two statistically significant cause-specific results (relative inequalities in cancer mortality among women and in mortality from other causes among men) also suggest that relative inequalities increased. We then added the trends for income inequality to the model to show, first, the effect of income inequality on mortality inequality and, second, whether the trend for mortality inequality changed once trends in income inequality were taken into account (Model 2 in Table 4).

Ignoring statistical significance, the coefficients for income inequality were mostly negative, suggesting that increasing income inequality was associated with decreasing mortality inequality. Five of 20 coefficients for the trend in income inequality were statistically significant: two negative coefficients for relative inequalities among women were observed (-0.36 and -1.19, respectively), which is against our hypothesis. Three positive coefficients were observed in line with our hypothesis: for mortality from external causes for both absolute and relative inequalities among women (0.01 and 0.83 respectively), and from CVD for relative inequalities among women (5.00). Overall, this suggests that increasing income inequality does not lead to increasing mortality inequality, but external causes and mortality from CVD among women may be an exception.

If we compare the annual trend in mortality inequality between Model 1 and Model 2, we see that, in most cases, the trend in mortality inequality improves (i.e., becomes less increasing, or more decreasing) if the trend in income inequality is taken into account. For external causes of death, all four trends are more favourable in Model 2 (which accounts for income inequality) than in Model 1. For example, in the first row (absolute inequalities among men) the annual trend of decreasing inequality of -0.12 becomes even more decreasing with -0.52 deaths per 100,000, although this trend was not statistically significant.

## Discussion

This study has shown that the general trend in income inequality between high and low educated people in the six countries increased during the study period while the mortality differences between educational groups showed diverse trends, with absolute differences mostly decreasing and relative differences increasing in some countries but not in others. A more detailed discussion of trends in mortality differences in Europe and its determinants has been published elsewhere [5]. We found no association between trends in income inequalities and trends in inequalities in all-cause mortality. Further, trends in mortality inequalities did not improve when adjusted for rising income inequalities. This result holds for absolute, as well as for relative, inequalities. A cause-specific analysis suggested some association for deaths from external causes, and to some extent also from cardiovascular diseases, but without statistical significance.

The strength of our study is that it used a substantive new data collection with comparable information on income and mortality by educational level over time and across countries. This approach goes beyond earlier studies that focussed on a single country [34] and could therefore not test a general link between income inequality and health inequality. Secondly, it goes beyond cross-sectional attempts to establish this link because it controls for country-specific fixed effects that might influence inequality in health or income. Such country-specific characteristics might be the setup of the welfare system or cultural values that are especially tolerant or adverse with regard to inequalities in health or income.

The longitudinal design was an advantage, but also a limitation to our study: the lack of comparable data over time, especially on income by education, made our time series shorter than we wanted and several trend estimations are based on only two time points. Income can be measured in many different ways, and many countries changed the way of measurement over time or used categorical measurement with too few categories which led to the exclusion of several countries and years. To respect temporal order between the income period starting first and the mortality period starting later further limited the overall amount of available data. We cannot exclude that the inconsistencies in the income measurement between countries influenced the results. For example, the results for England & Wales and Denmark are based on gross incomes, thus the income inequalities are biased upwards. However, the increase in income inequality might be biased downwards, at least in England & Wales where substantial reductions in the tax rates for top income levels were introduced in the observed period. While this limits the comparability of the level and the trend of income inequalities, it is unlikely to create a systematic bias that influenced the answer to our research question, because the trend of income inequality in England & Wales is already steeply increasing using the available income measures. A second limitation is that mortality data and income data come from different sources and can only be matched through the assumption that the samples are representative for the same population. While our mortality either covers whole national populations or stems from 1 % representative samples, our income data comes from health surveys that usually suffer from a participation bias in favour of more educated and higher-income people. Thus, income inequalities in our study might be underestimated, but that does not necessarily imply that trends in income inequality are biased. Only the latter bias would have an effect on our results. To solve this data problem, register data with mortality and income from the same persons over time could be used, but it only exists in very few Scandinavian countries. Third, this data situation implied that we could only use only a small number of countries, which may account for the low level of statistical significance of our results. Fourth, differences between countries in classification of causes of death could also have affected our results. For example, certification and coding of ischemic heart disease vary between countries, and a substantial underestimation of ischemic heart disease in official mortality statistics has been reported for France [48]. Even if such underestimation does not differ between socioeconomic groups, and does not affect estimates of relative inequalities in mortality, it will affect estimates of absolute inequalities in mortality from ischemic heart disease. Similar problems may be present for other causes of death. However, as our analysis focused on changes over time within countries, between-country variations in data collection do not pose a major risk of bias. Generally, we consider mortality to be a good and objective indicator for health. But it is noteworthy that alternative health measures, such as self-rated health, might have revealed higher responsiveness to changes in income, be it because it reacts faster than mortality, or because it captures more subjective feelings about well-being. Future research should verify whether our findings also apply to alternative health measures. Finally, we compared low- and high-educated people, leaving out the middle educated group, in order to use only two very different groups and observe their differences over time. The comparison of three groups is much more complex and dividing the whole population in two educational groups implies difficult compromises in the categorization of mid-educated people that would have decreased rather than increased international comparability. The excluded mid-educated population ranges between 25.4 % (England & Wales in 1990) and 66.2 % (Switzerland in 2002) (Table 1). This difference in size mirrors the difference in size of the groups included in our study and smaller educational groups at the very end of the educational distribution are likely to be more selected and produce more extreme results. However, changes in educational group sizes over time are much smaller than differences between countries, so we assume that our trend analysis was not biased substantially.

Our research question contributes to the larger discussion on the reasons for changes in health inequality over time. Several contributing factors have been discussed: changes in social inequality [2] or changes in the health returns of a certain socioeconomic status [49], changes in the social distribution of more proximate (behavioural) risk factors [21], and changes in the distribution of health care [50, 51]. These are also the factors that may explain international differences in changes in health inequalities. Our findings suggest that changes in income inequality have only minor effects on health inequality. This could be because income, relative to many other determinants of health, is not important enough to show a clear determination on health in our study design because it is not a strong mediator between education and health. This argument may be especially valid in a sample of relatively rich countries where, despite increasing income inequalities, low-educated people may not have experienced absolute income losses. Income is an important predictor for health on the individual level, but it is possible that much of its predictive power comes from associations (with working conditions, health behaviour, health care) rather than from causal effects of income on health-relevant material living conditions. This argument includes the possibility that causality partly goes from health to income [52].

It is also possible that our aggregated data leads to ecological biases where distributional changes over time in the involved variables or in their associations hide the actual association.

Finally, a lack of association could be due to the fact that trends in inequalities in mortality are strongly determined by the progression of the smoking epidemic. Our findings support the view that the development of health inequalities is slightly more positive for men than for women, which has been attributed to women being in an earlier stage of the smoking epidemic [33, 53–55]. In many fields of mortality analysis, it is now common practice to “remove” the effects of smoking before other influences are studied. As a sensitivity analysis, we used the Preston-Glei-Wilmoth method [56] to calculate non-smoking attributable mortality by level of education. We established the mortality trends for non-smoking related mortality and calculated the regression results in Table 4 also for this subset of mortality (see Additional file 3: Table S3). The results show that excluding smoking related causes of death changed the results for women: trends in mortality inequality were better for non-smoking related causes of death than for all-cause mortality. However, the results lead to the same conclusion with regard to our research question: first, most coefficients for the effect of income inequality on mortality inequality are negative, and none is statistically significant; second, most trends of mortality inequality get worse once income inequality is controlled for. This suggests that smoking does not influence our main findings.

While all-cause mortality was unrelated to income inequality, a cause specific-analysis revealed two causes of death for which educational inequalities seem to depend on income inequality. Although our findings were not statistically significant, it is plausible and in line with previous findings that mortality from external causes, such as accidents, violence and suicide, and CVD depend more on socioeconomic determinants than cancer and other causes of death [5]. Cancers as a whole are less associated with socioeconomic determinants because some specific cancers are unrelated or inversely associated with socioeconomic position. A simple explanation is that for some cancers (e.g. breast and prostate cancer) it is not clear how they can be prevented, so there is less advantage for people with high socioeconomic status than in the cases of CVD, where knowledge and healthy behaviour can prevent disease, and external causes that also largely depend on general social living conditions [57]. It is noteworthy that our analysis did not include deaths from external causes that occur below age 35, because education as an indicator is less useful at young ages. A detailed analysis of external causes of death among young people might provide additional insights.

A second reason may explain the difference between the findings for all-cause mortality and the cause-specific results. In general, there is little knowledge about the time lag in which mortality reacts to changes in income. Two recent studies with good data and methods reveal an effect of income inequality after 5 to 12 years for people aged 30+ [58] and a life-long effect of income inequality in childhood [59]. Income inequality works through different mechanisms than income, but the timing chosen in our study, which is largely determined by data availability, may not cover time lags long enough to observe the effect of changes in income. In this regard, it is plausible that causes of death that react faster to changes in the economic status, such as external causes and potentially CVD, show the expected association that was not observed for all-cause mortality.

## Conclusions

We did not find consistent evidence that trends in income inequality between educational groups are associated with or predict trends in mortality differences between educational groups. Possible reasons for the overall lack of the assumed association could be, first, that income is not a sufficiently important determinant of mortality, or at least does not mediate to a large extent the effect of education on mortality in the setting of high income countries. Second, it is possible that our assumed time-lag between changes in income and mortality does not sufficiently reflect different time-lags for different causes of death. Future research should look at differences when using health measures other than mortality and at differences between causes of death using even more specific causes of death to further test our overall hypothesis. A decisive step forward can probably be done only with large, longitudinal individual-level data sets from a range of different countries with information on mortality and income for the same persons, which is currently unavailable.

## Abbreviations

CVD, cardiovascular diseases; ISCED, International Standard Classification of Education; PPP, purchasing power parities; RD, rate differences; RR, rate ratios.

## Additional files

Additional file 1: Table S1. Annual changes of absolute and relative inequalities in cause-specific mortality. (DOCX 14 kb) Additional file 2: Table S2. Correlation coefficients between country-specific trends in inequality in mortality and country-specific trends in income inequality. (DOCX 12 kb) Additional file 3: Table S3. Sensitivity analysis comparing all-cause and non-smoking related mortality. (DOCX 12 kb)
