# Supplementary material for: The impact of increasing income inequalities on educational inequalities in mortality - An analysis of six European countries
Source: Int J Equity Health. 2016 Jul 8;15:103. doi: 10.1186/s12939-016-0390-0 (PMC4938956; doi:10.1186/s12939-016-0390-0)
Supplement: Additional file 1: Table S1. — Annual changes of absolute and relative inequalities in cause-specific mortality. (DOCX 14 kb) [file 12939_2016_390_MOESM1_ESM.docx]

**Additional Table 1: Annual changes of absolute and relative inequalities in cause-specific mortality**

|  | **CVD** | | | | **CANCER** | | | | **EXTERNAL CAUSES** | | | | **OTHER** | | | |
| --- | --- | --- | --- | --- | --- | --- | --- | --- | --- | --- | --- | --- | --- | --- | --- | --- |
|  | **absolute** | | **relative** | | **absolute** | | **relative** | | **absolute** | | **relative** | | **absolute** | | **relative** | |
|  | men | women | men | women | men | women | men | women | men | women | men | women | men | women | men | women |
| Belgium | N.A. | N.A. | N.A. | N.A. | N.A. | N.A. | N.A. | N.A. | N.A. | N.A. | N.A. | N.A. | N.A. | N.A. | N.A. | N.A. |
| Denmark | -6.33 | -6.05 | 0.72 | -3.95 | 8.29* | 6.81 | 3.27** | 2.76 | -0.13 | 0.82 | 5.16 | 3.58 | 8.12 | 7.35 | 6.17 | 6.42 |
| E & W | -6.07** | **-**2.59 | 1.24 | 1.47 | -2.56 | 0.38 | -0.13 | 0.64 | 0.03 | 1.04 | 0.49 | 0.43 | 1.93 | -0.36 | 2.80 | -1.53 |
| France | -7.48 | -1.87 | -8.56 | -6.42 | -2.02 | 6.76 | -0.57 | 4.07 | -2.74 | -0.57 | -19.37 | -0.07 | -2.62 | 0.86 | -2.21 | 2.55 |
| Slovenia | 21.79 | -32.31 | 12.42 | 17.01 | 10.12 | 4.90 | 4.48* | 2.58 | N.A. | N.A. | N.A. | N.A. | 0.59 | 2.08 | 7.23 | 8.01** |
| Switzerland | -3.85 | -3.12* | 3.53** | 0.29 | -1.26 | 2.14 | 0.99 | 1.60 | 0.11 | -0.35 | 3.15** | -1.35 | -4.58 | 1.77 | 2.23 | 3.68 |

Note: Belgium did not provide mortality data by cause of death and Slovenia provided no data on external causes
