# Supplementary material for: The impact of increasing income inequalities on educational inequalities in mortality - An analysis of six European countries
Source: Int J Equity Health. 2016 Jul 8;15:103. doi: 10.1186/s12939-016-0390-0 (PMC4938956; doi:10.1186/s12939-016-0390-0)
Supplement: Additional file 2: Table S2. — Correlation coefficients between country-specific trends in inequality in mortality and country-specific trends in income inequality. (DOCX 12 kb) [file 12939_2016_390_MOESM2_ESM.docx]

**Additional Table 2: Correlation coefficients between country-specific trends in inequality in mortality and country-specific trends in income inequality**

|  |  | **TOTAL MORTALITY** | **CVD** | **CANCER** | **EXTERNAL** | **OTHER** |
| --- | --- | --- | --- | --- | --- | --- |
| **Absolute inequality** | men | -0.33 | -0.47 | -0.23 | 0.91* | 0.42 |
|  | women | -0.78* | 0.54 | -0.61 | 0.90* | 0.07 |
| **Relative inequality** | men | -0.27 | -0.25 | -0.35 | 0.90 | -0.01 |
|  | women | -0.86** | 0.87* | -0.62 | 0.41 | -0.62 |
