# Supplementary material for: The impact of increasing income inequalities on educational inequalities in mortality - An analysis of six European countries
Source: Int J Equity Health. 2016 Jul 8;15:103. doi: 10.1186/s12939-016-0390-0 (PMC4938956; doi:10.1186/s12939-016-0390-0)
Supplement: Additional file 3: Table S3. — Sensitivity analysis comparing all-cause and non-smoking related mortality. (DOCX 12 kb) [file 12939_2016_390_MOESM3_ESM.docx]

**Additional Table 3: Sensitivity analysis comparing all-cause and non-smoking related mortality**

|  |  |  | **TOTAL MORTALITY** | | **NON-SMOKING RELATED MORTALITY** | |
| --- | --- | --- | --- | --- | --- | --- |
|  |  |  | Model 1 | Model 2 | Model 1 | Model 2 |
| Absolute inequalities | men | annual trend | -6.87 | -6.83 | -1.68 | -10.15 |
|  |  | income inequality |  | -0.01 |  | 0.08 |
|  | women | annual trend | -0.73 | 2.41 | -8.13* | -0.78 |
|  |  | income inequality |  | -0.05* |  | -0.13 |
| Relative inequalities | men | annual trend | 1.31 | 1.30 | 0.89 | 0.98 |
|  |  | income inequality |  | -0.18 |  | -0.06 |
|  | women | annual trend | 1.07 | 1.71** | -1.72 | -0.57 |
|  |  | income inequality |  | -0.45** |  | -0.95 |

Note: the setup of the models is the same as in the main result Table 4. The results for total mortality differ from Table 4 because Belgium was excluded from this sensitivity analysis due to the lack of cause-specific mortality data: non-smoking related mortality could not be defined.
